# Supplementary material for: Characterization of bacterial intrinsic transcription terminators identified with TERMITe—a novel method for comprehensive analysis of Term-seq data
Source: Nucleic Acids Res. 2025 Jun 30;53(12):gkaf553. doi: 10.1093/nar/gkaf553 (PMC12207403; doi:10.1093/nar/gkaf553)
Supplement: gkaf553_Supplemental_Files [file gkaf553_supplemental_files.zip › Supplementary methods.pdf]

SUPPLEMENTARY METHODS

Characterization of bacterial intrinsic transcription terminators identified with TERMITe – a novel method for comprehensive analysis of Term-seq data

Jan Grzegorz Kosiński, Sandeepani Ranaweera, Agnieszka Chełkowska-Pauszek, Mikhail Kashlev, Paul Babitzke and Marek Żywicki

Gblock and oligonucleotides used to generate templates for in vitro transcription. In each gene block the in vivo point of termination is in bold type and the positions of oligonucleotide hybridization are underlined.

cysZ gblock

5′ AATACTGAATTGTAAGAATTCAAGAACAGCTTGACAAATACACAAGAGTGTGTTATAATG
CAATTAGAATGAGTTGAGTTAGAGAATAGTATTGCGATTACCCCTTCGATAACCACAAAG
TGCCGTTTAAAGAGATGCGCACCGCCCTGCGCACACGCAAAATCACCAATATGCAGTTTG
GTGCTTTAACCAGCCTGTTTACGATGATCCCGCTGCTTAATCTGTTCATCATGCCCGTTG
CCGTTTGTGGCGCGACGGCGATGTGGGTCGATTGCTATCGCGATAAACACGCGATGTGGC
GGTAACAATCTACCGGTTATTTTGTAAACCGTTTGTGTGAAACAGGGGTGGCTTATGCCG
CCCCTTAT**T**CCATCTTGCATGTCATTATTTCCCAAGCTTGGCACTGGCCGTCGTTTTACA
ACGTCGTGA 3′

yajD gblock

5' AATACTGAATTGTAAGAATTCAAGAACAGCTTGACAAATACACAAGAGTGTGTTATAATG
CAATTAGAATGAGTTGAGTTAGAGAATCAGTATGGTACGACCGTTATCGCAGGGGAAGAT
GCGCAGAAAGATGTCGGTGAAGCGAAGTACAACCCATTCGCTGACCTGAAAGCGATGATG
AACAAGAAGAAGTGATTAAAACGTAAAATTGCCTGATGCGCTACGCTTATCAGGCCTACG
TTATTTCAGCAATATATTGAATTTACGTGCTTTTGTTGGCCGGACAAAGCGTTTACGCCG
CATCCGGCATGAACAAAGCACACGTTGTTAACAATCAGAAATGCCGGGAATAAATCCCGG
CATTTTCA**T**AATCAGAAGTTGTAACCTACTACCAAGCTTGGCACTGGCCGTCGTTTTACA
ACGTCGTGA 3'

yibL gblock

5′ AATACTGAATTGTAAGAATTCAAGAACAGCTTGACAAATACACAAGAGTGTGTTATAATG
CAATTAGAATGAGTTGAGTTAGAGAATATTCCAGCGCGCAATTACCAAAAAAGAGCAGGC
TGATATGGGCAAGCTGAAGAAAAGTGTTCGCGGACTGGTCGTTGTGCACCCAATGACCGC
ACTGGGCCGCGAAATGGGCCTGCAGGAGATGACTGGGTTTTCAAAGACCGCGTTTTAAGA
ACACAGTATCTACAGGGTGATTCTGCACATTCCTATAGGCCGAGTAAGGTGTTCACGCCG
CATCCGGCAAGATAAGGCGCTCTGGATCAACAACCTAAGGGCAATTCTCTGATGAGGATT
GCCCTTTTC**T**TTACCAGACATCTCCCCCCACAAAAGCTTGGCACTGGCCGTCGTTTTACA
ACGTCGTGAC 3′

sgrS gblock

5′ AATACTGAATTGTAAGAATTCAAGAACAGCTTGACAAATACACAAGAGTGTGTTATAATG
CAATTAGAATGAGTTGAGTTAGAGAATGATGAAGCAAGGGGGTGCCCCATGCGTCAGTTT
TATCAGCACTATTTTACCGCGACAGCGAAGTTGTGCTGGTTGCGTTGGTTAAGCGTCCCA
CAACGATTAACCATGCTTGAAGGACTGATGCAGTGGGATGACCGCAATTCTGAAAGTTGA
CTTGCCTGCATCATGTGTGACTGAGTATTGGTGTAAAATCACCCGCCAGCAGATTATACC
TGCTGGTTTTTTT**T**ATTCTCGCCGCGCTAAAAAGGGAAGCTTGGCACTGGCCGTCGTTTT
ACAACGTCGTGA 3′

rybB gblock

5′ AATACTGAATTGTAAGAATTCAAGAACAGCTTGACAAATACACAAGAGTGTGTTATAATG
CAATTAGAATGAGTTGAGTTAGAGAATGCCACTGCTTTTCTTTGATGTCCCCATTTTGTG
GAGCCCATCAACCCCGCCATTTCGGTTCAAGGTTGATGGGTTTTTG**T**TATCTAAAACTT
ATCTACTAAAGCTTGGCACTGGCCGTCGTTTTACAACGTCGTGAC 3′

TermSGB_Fw oligonucleotide

5′ GTAAGAATTCAAGAACAGCTTGACAAATACACAAGAGTGTG 3′

TermSGB_RV oligonucleotide

5′ CGTTGTAAAACGACGGCCAGTGCCAAGC 3′
