# Supplementary material for: Characterization of bacterial intrinsic transcription terminators identified with TERMITe—a novel method for comprehensive analysis of Term-seq data
Source: Nucleic Acids Res. 2025 Jun 30;53(12):gkaf553. doi: 10.1093/nar/gkaf553 (PMC12207403; doi:10.1093/nar/gkaf553)
Supplement: gkaf553_Supplemental_Files [file gkaf553_supplemental_files.zip › Supplemetary figures and tables.pdf]

SUPPLEMENTARY FIGURES AND TABLES

Characterization of bacterial intrinsic transcription terminators identified with TERMITe – a novel method for comprehensive analysis of Term-seq data

Jan Grzegorz Kosiński, Sandeepani Ranaweera, Agnieszka Chełkowska-Pauszek, Mikhail Kashlev, Paul Babitzke and Marek Żywicki

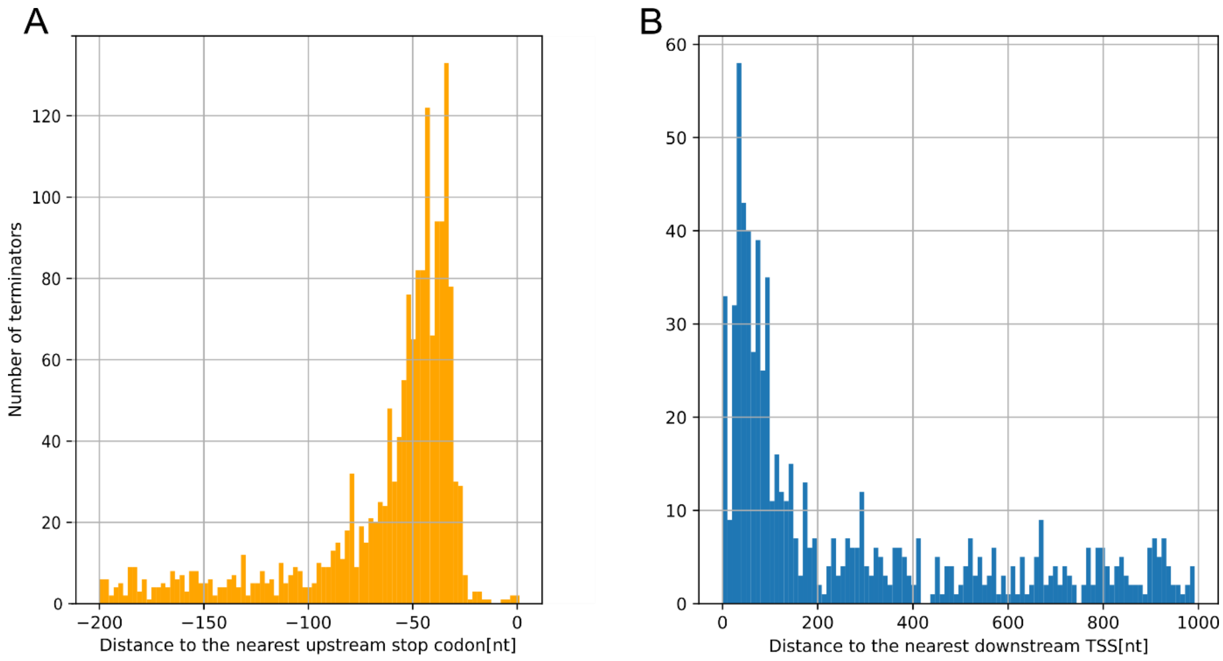

Supplementary Figure 1. Distribution of distances from identified intrinsic termination sites to closest genes. **A)** Distances to nearest upstream stop codon. **B)** Distances to nearest downstream transcription start site.

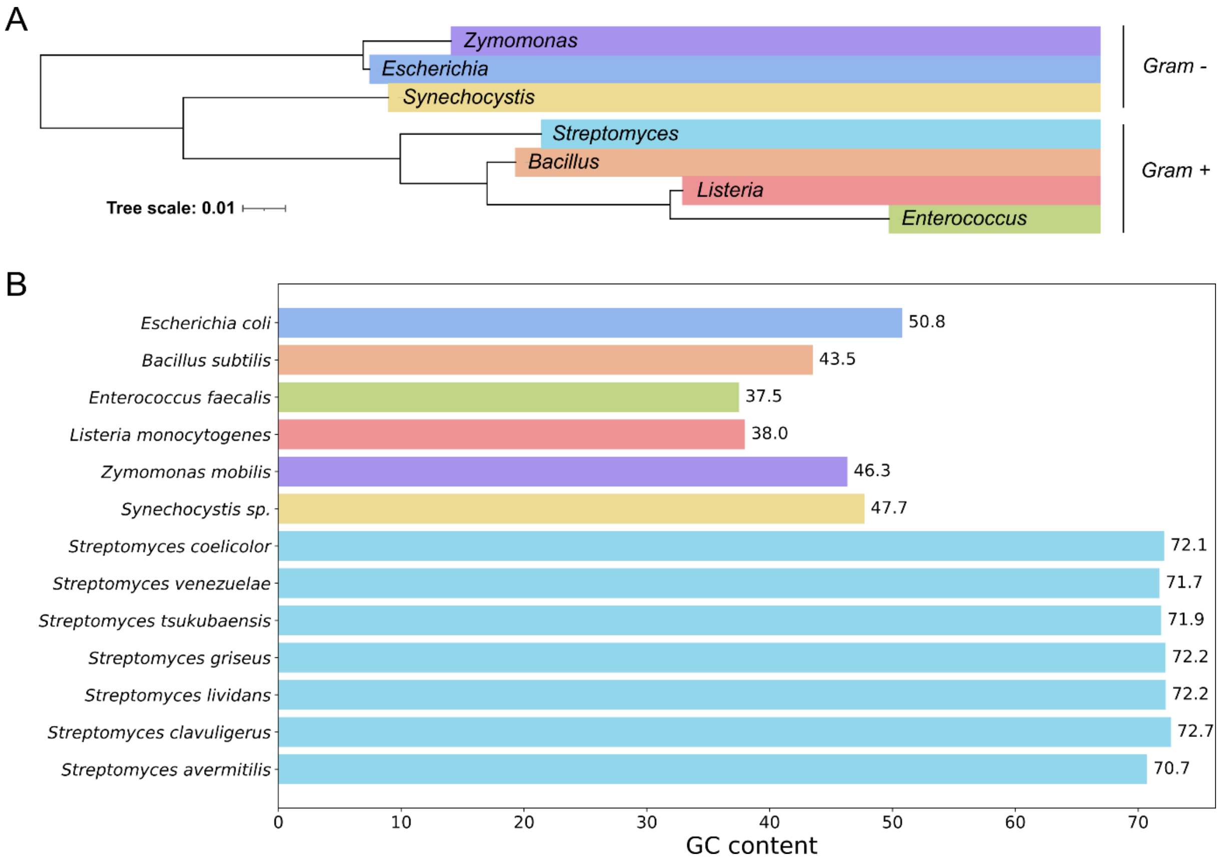

Supplementary Figure 2. A) Phylogenetic tree of the analyzed genera. Gram-positive bacteria are marked with (+), while Gram-negative with (-). The phylogenetic tree was visualized using *iTOL v6* (Letunic and Bork, 2021). See Materials and Methods for detailed description of the visualization procedure. The lengths of the edges correspond to the evolutionary distances between genera**. B)** %GC content of the analyzed reference genomes (plasmid sequences were not considered).

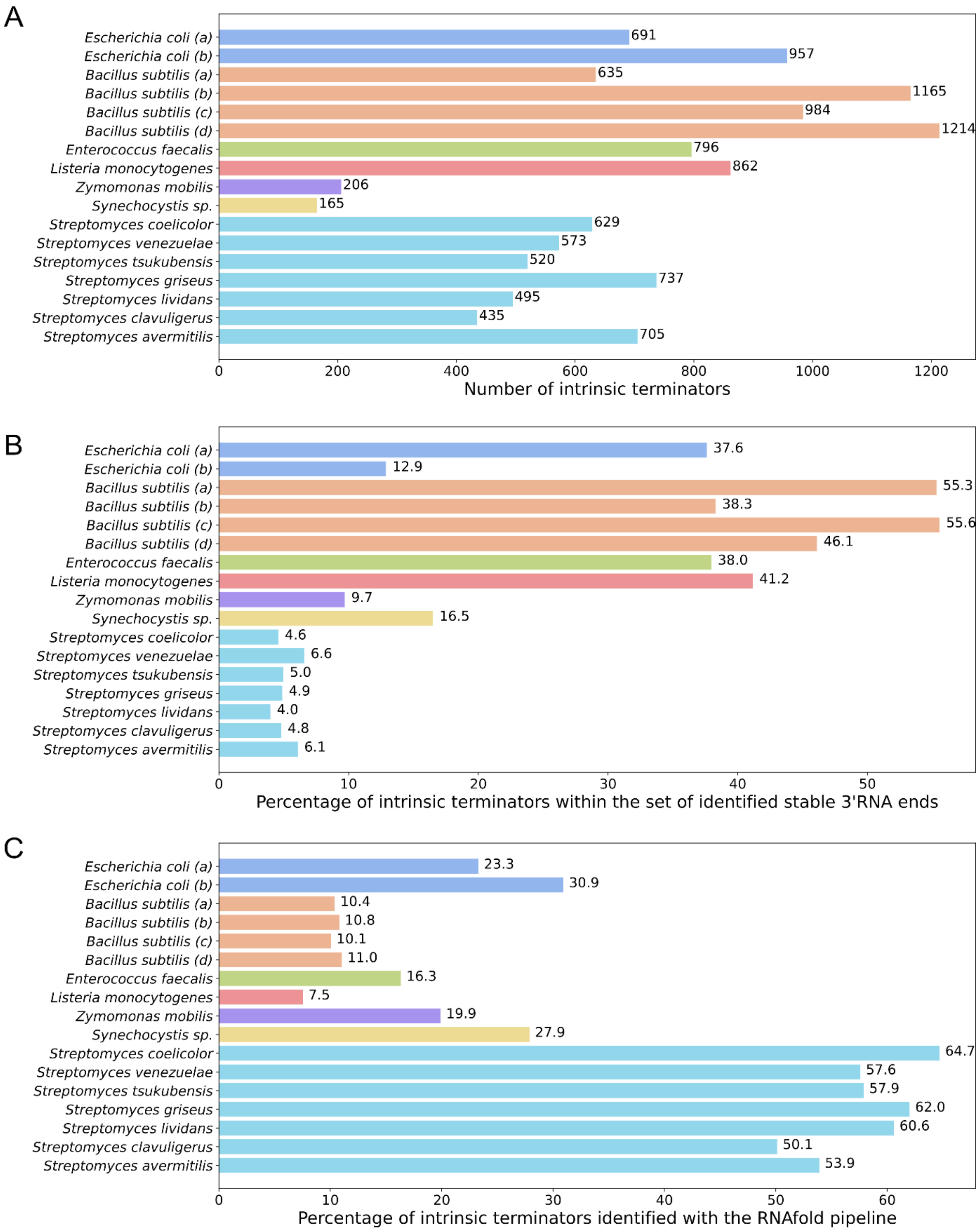

Supplementary Figure 3. Summary of intrinsic termination events identified by TERMITe**. A)** The number of identified intrinsic transcription terminators. **B)** The percentage of the stable 3' ends reported as intrinsic terminators for each species and experiment. **C)** The percentage of intrinsic terminators identified solely with the RNAfold pipeline.

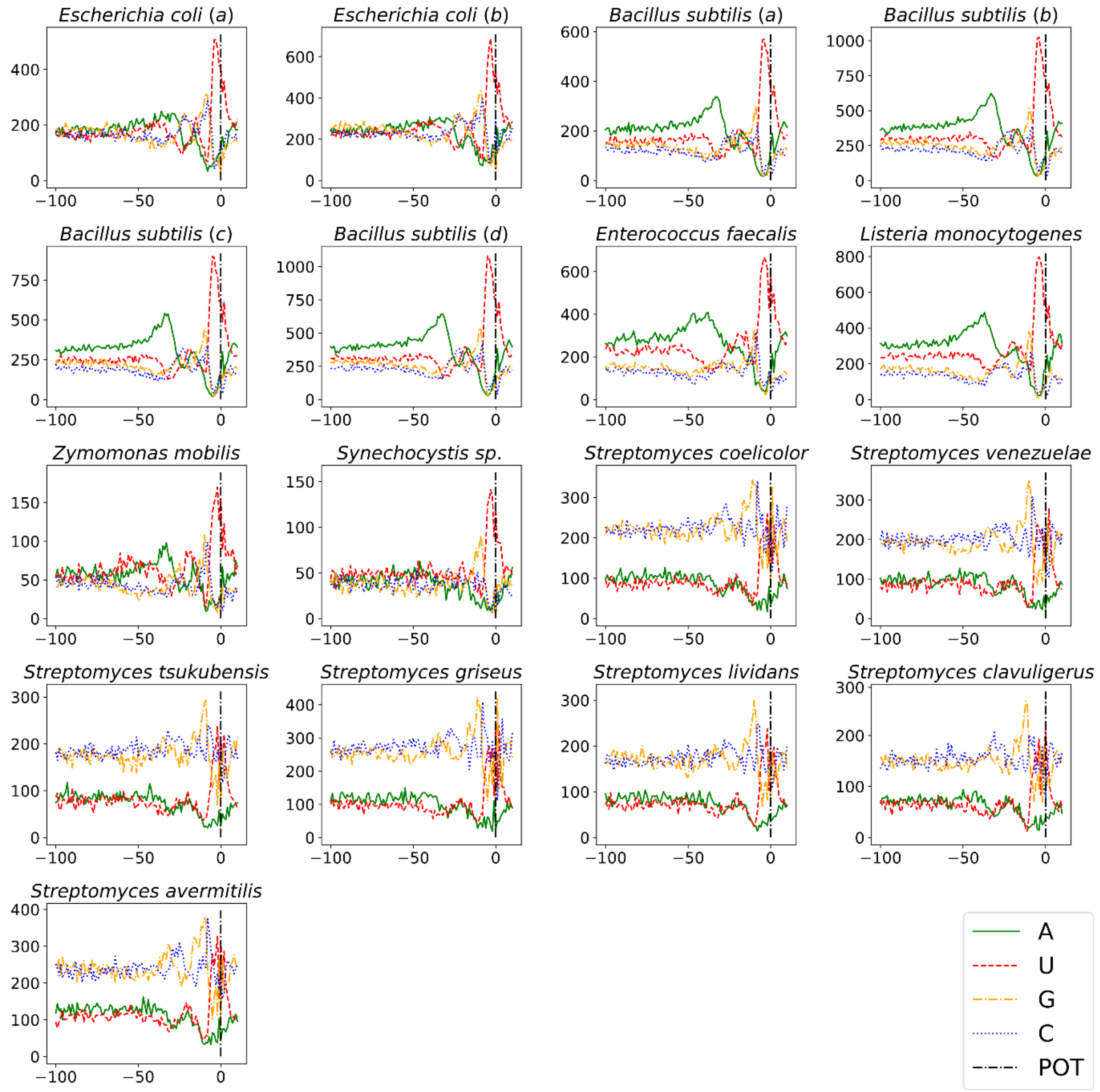

Supplementary Figure 4. Nucleotide composition of the sequence region starting 100 nt upstream and ending 10 nt downstream from the POT (position 0) for all identified intrinsic terminators in each experiment. The black vertical line marks the POT.

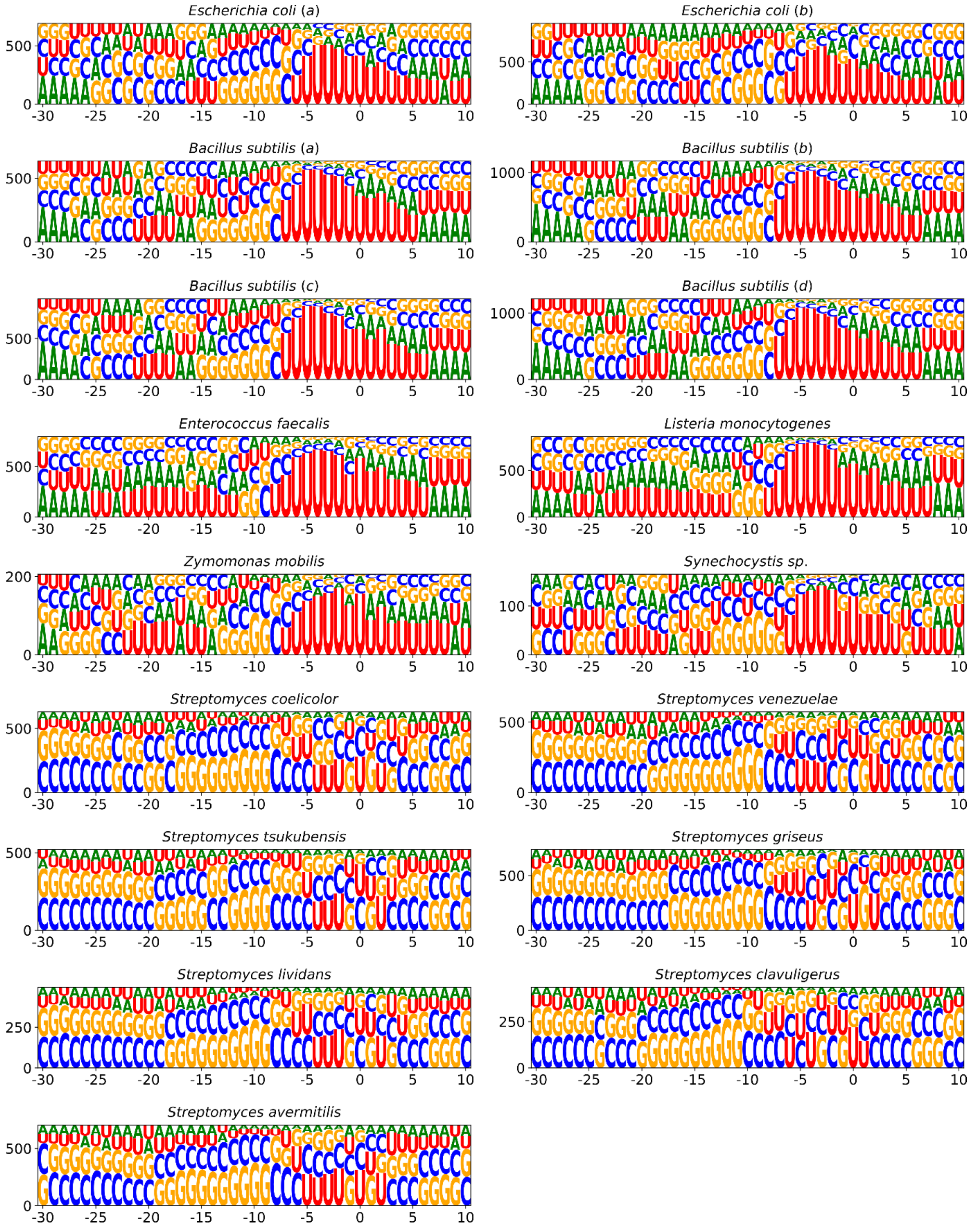

Supplementary Figure 5. Sequence logos showing the nucleotide compositions of the terminator regions (30 nt upstream and 10 nt downstream from the POT) for all identified intrinsic terminators in each experiment. The POT is at position 0.

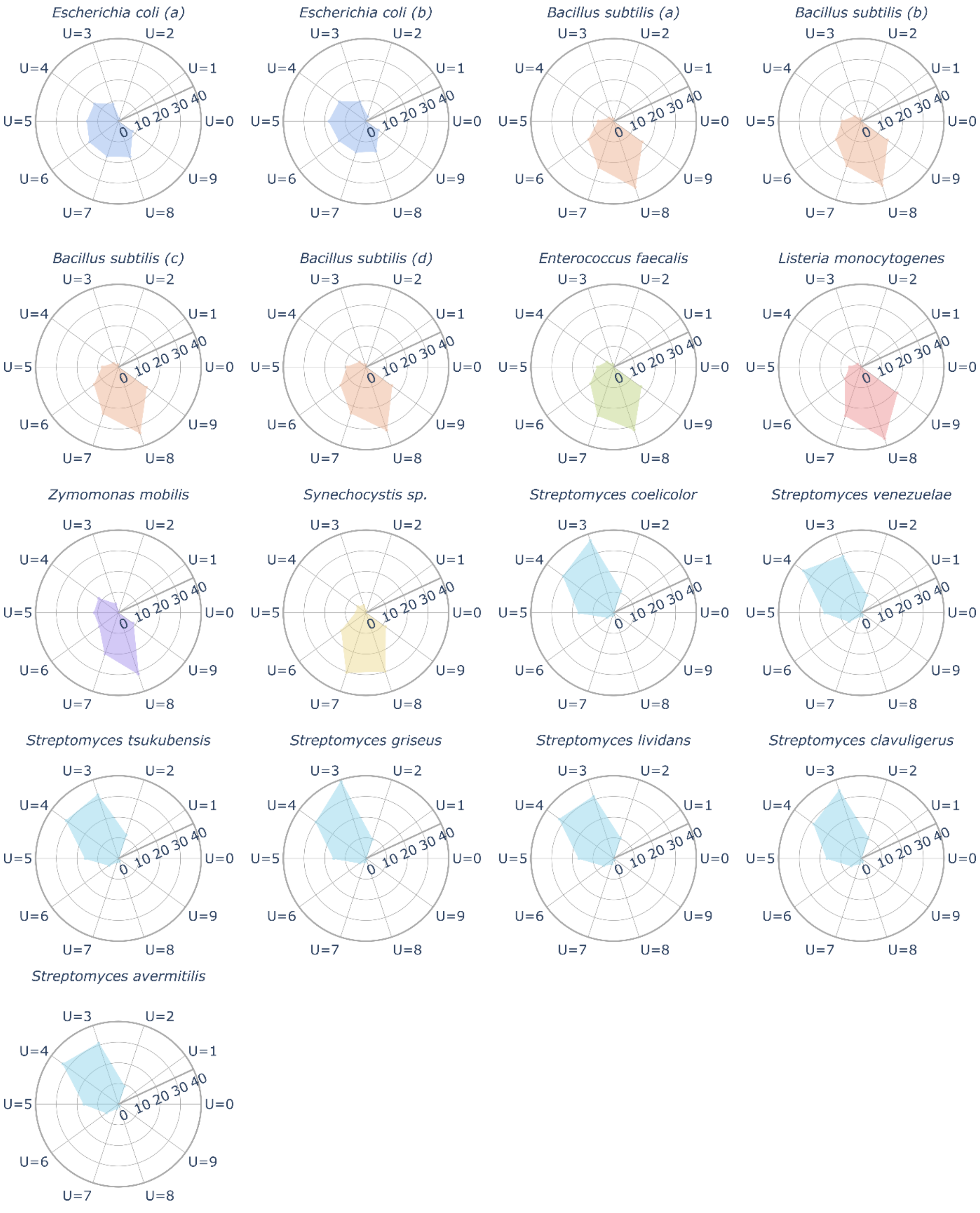

Supplementary Figure 6. The percentage of the U tracts (defined as 10 nucleotides immediately downstream from the hairpin base) containing the specified number of U nucleotides within all identified intrinsic terminators in each experiment.

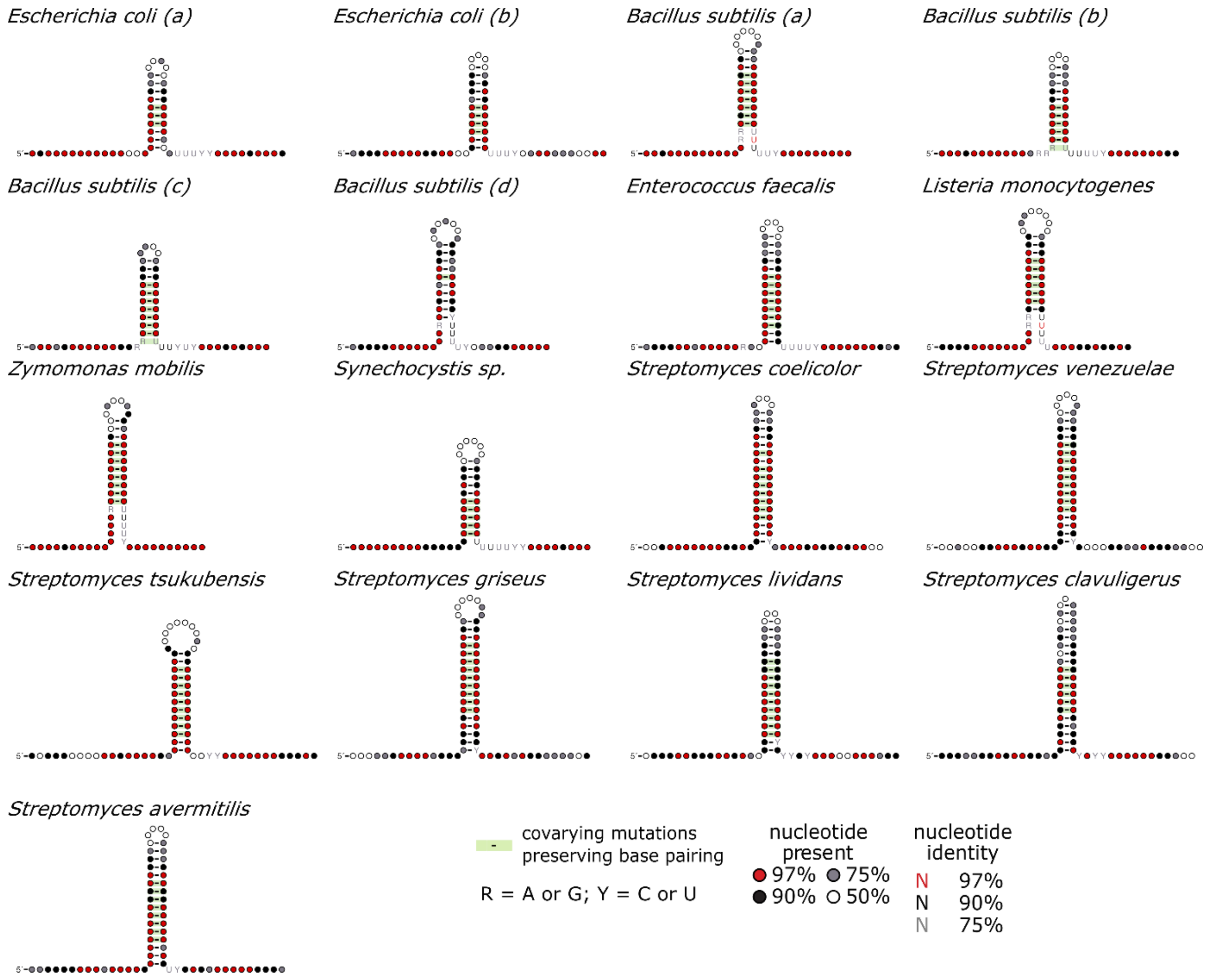

Supplementary Figure 7. The consensus secondary structure of all identified intrinsic terminator hairpins along with the information about nucleotide conservation. The base-pairing between the U-rich tract and the 5’ region of the hairpin was unmarked manually. See detailed description can be found in Figure 3 in the main text.

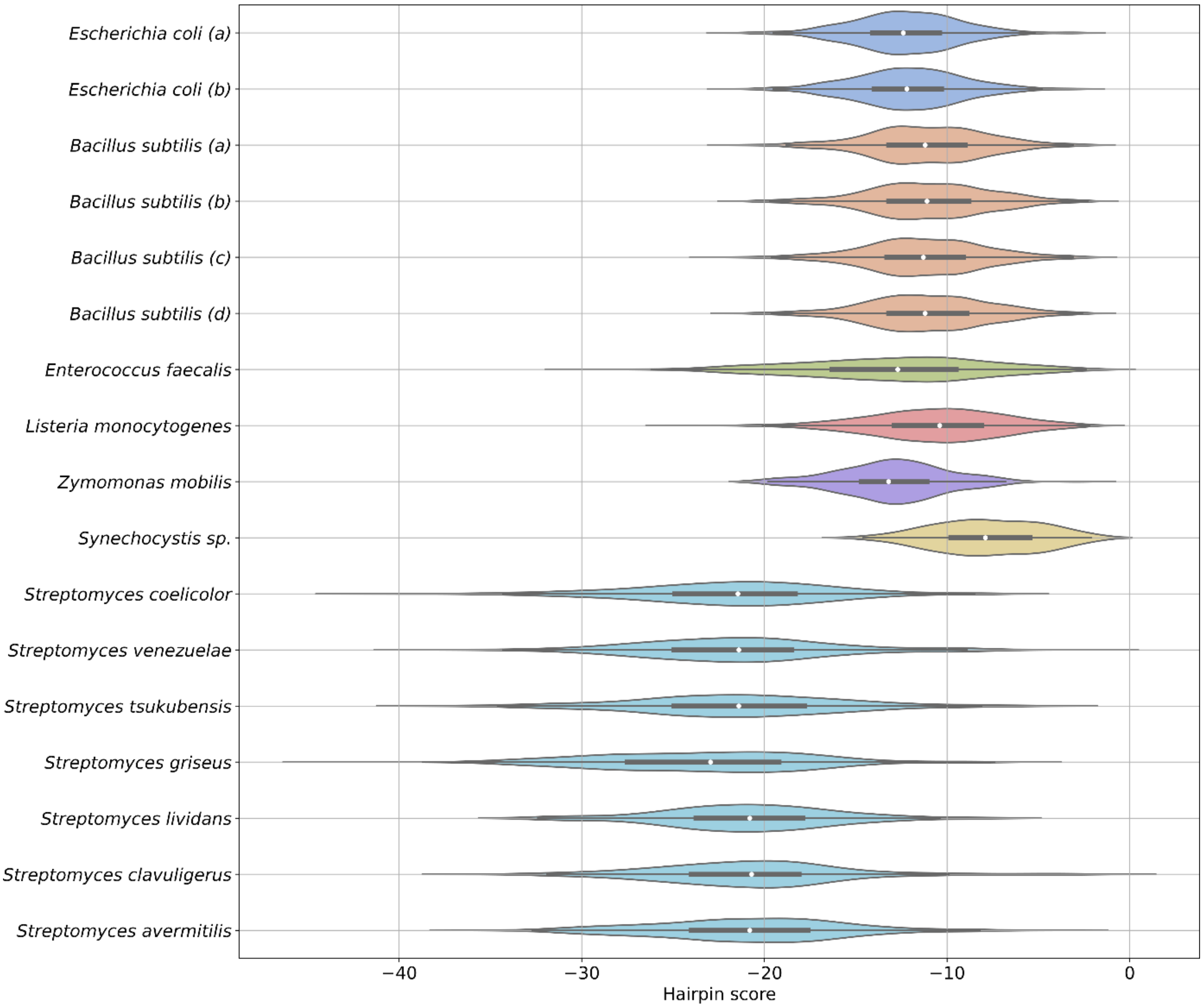

Supplementary Figure 8. Distribution of hairpin scores calculated by the TransTermHP pipeline for all identified intrinsic terminators in each dataset. Medians has been marked with white circle. The statistical parameters of distributions are provided in Supplementary Table 5.

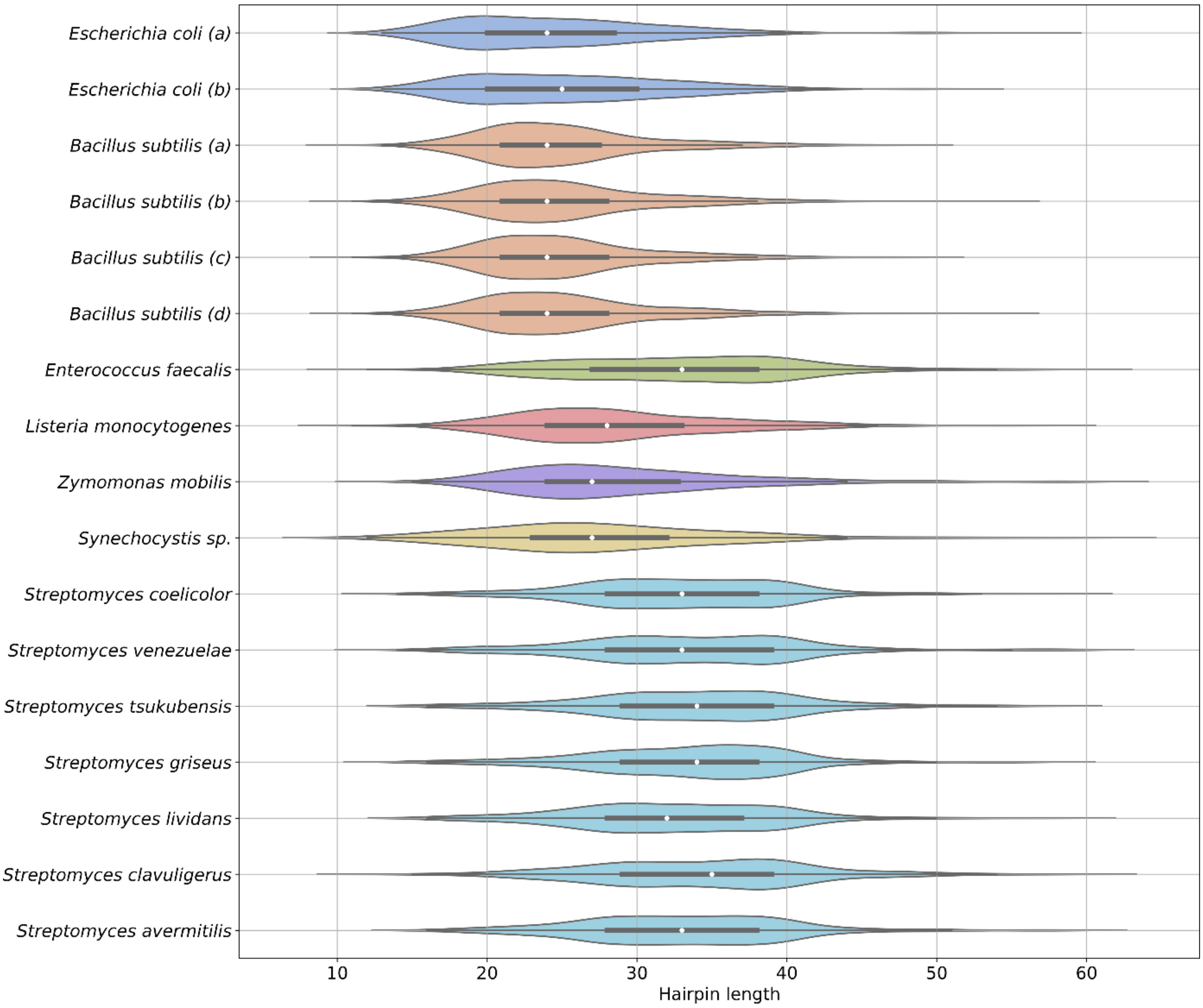

Supplementary Figure 9. Distribution of hairpin lengths of all identified intrinsic terminators in each experiment. The hairpin length is defined as the number of nucleotides involved in the hairpin formation. Medians has been marked with white circle. The statistical parameters of distributions are provided in Supplementary Table 6.

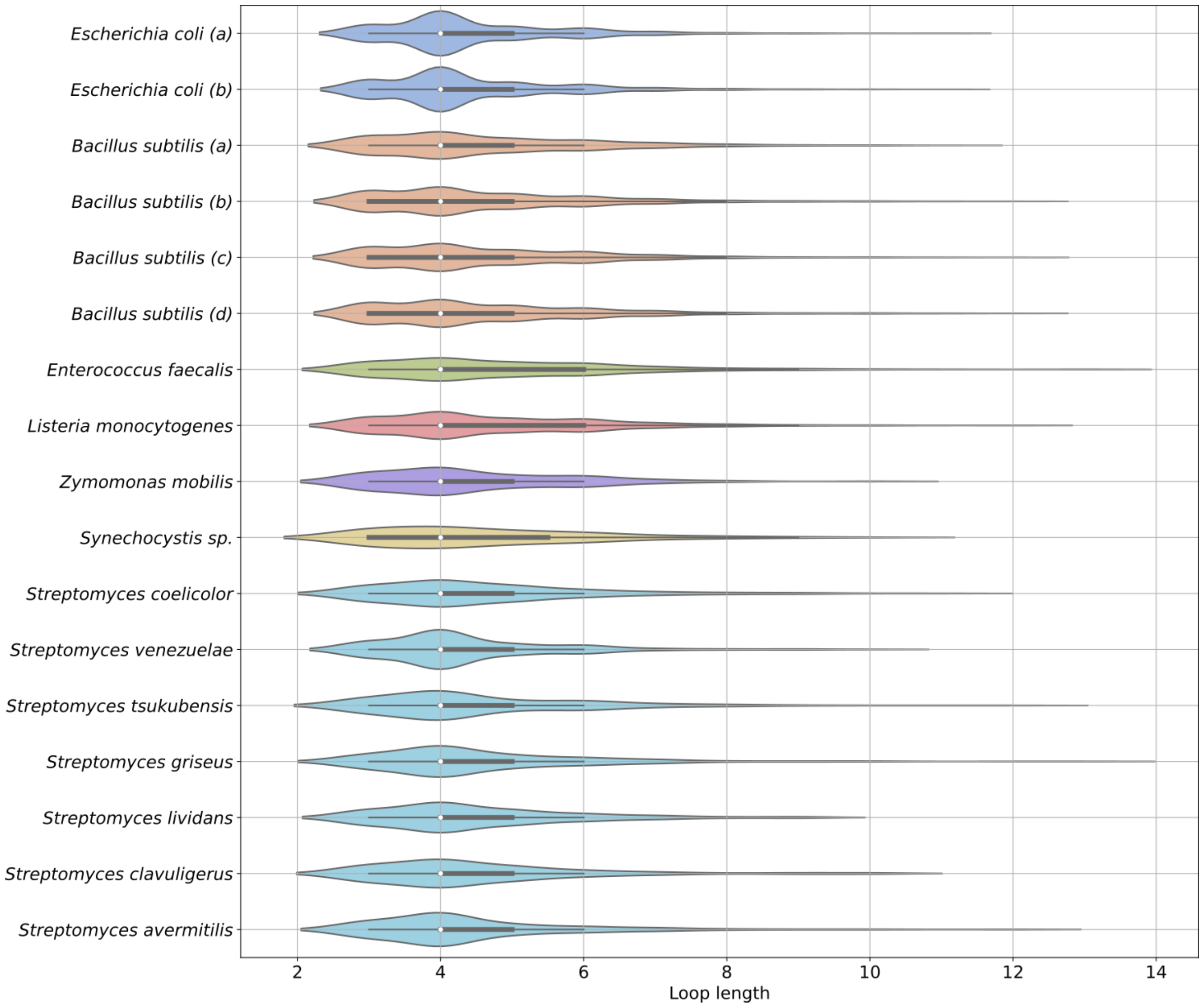

Supplementary Figure 10. Distribution of hairpin loop lengths of all identified intrinsic terminators in each analyzed dataset. Medians has been marked with white circle. The statistical parameters of distributions are provided in Supplementary Table 7.

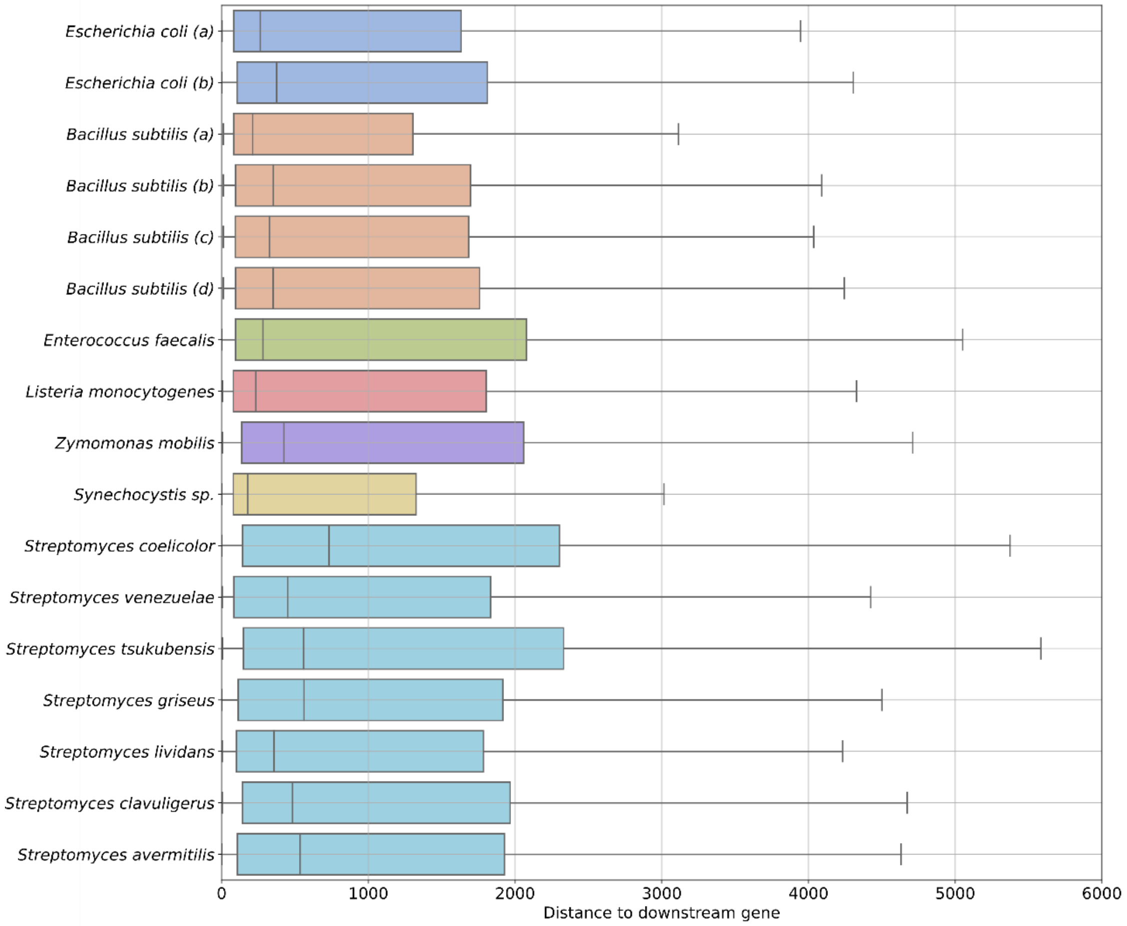

Supplementary Figure 11. Distributions of the distances between the POT and the nearest downstream CDS located on the same strand. Distributions are presented separately for each analyzed dataset. The statistical parameters of distributions are provided in Supplementary Table 11.

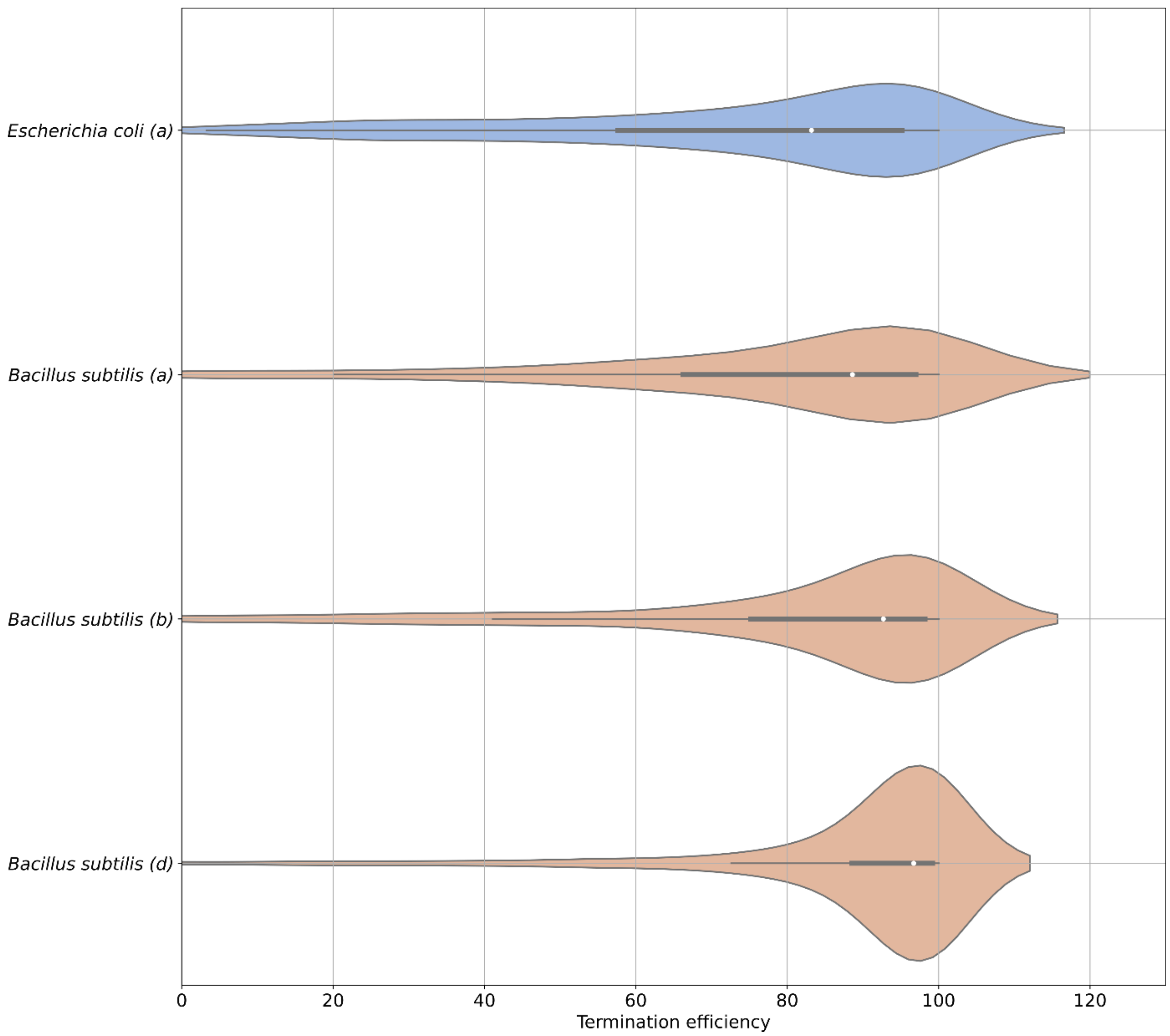

Supplementary Figure 12. Distribution of termination efficiencies calculated for all identified intrinsic terminators in each analyzed dataset prepared by the Term-seq protocol published by Mondal et al. Medians has been marked with white circle. The statistical parameters of distributions are provided in Supplementary Table 12.

Supplementary Table 1. Description of Term-seq datasets used in the study. [Table provided as separate Excel file, available online]

Supplementary Table 2. The complete set of intrinsic terminators identified in the study. [Table provided as separate Excel file, available online]

Supplementary Table 3. Descriptive statistics calculated to characterize distributions of hairpin Minimum Free Energy (MFE) values of all identified intrinsic terminators in each dataset. Abbreviations: *nobs*, number of observations; *Q1*, first quartile; *Q3*, third quartile; *min*, minimum; *max*, maximum; *mean*, arithmetic mean; *variance*, unbiased variance (*nobs -1* was used as a denominator); *skewness*, distribution skewness with denominator equal to *nobs*; *kurtosis*, normalized kurtosis of the distribution (kurtosis = 0 for the normal distribution), no degrees of freedom were used for the calculations.

	nobs	Q1	median	Q3	min	max	mean	variance	skewness	kurtosis
Escherichia coli (a)	650	-18.3	-15.2	-12.2	-28.9	-4.9	-15.29	17.65	-0.22	-0.05
Escherichia coli (b)	904	-18.6	-15.2	-12.08	-28.5	-3.9	-15.36	18.74	-0.22	-0.44
Bacillus subtilis (a)	564	-16.3	-13.5	-11.4	-27.9	-5.6	-13.94	14.19	-0.55	0.32
Bacillus subtilis (b)	1005	-16.5	-13.4	-11.0	-27.9	-4.0	-13.77	15.84	-0.36	0.15
Bacillus subtilis (c)	867	-16.5	-13.7	-11.5	-27.9	-4.1	-13.99	14.36	-0.43	0.25
Bacillus subtilis (d)	1053	-16.4	-13.4	-11.2	-27.9	-4.0	-13.8	15.17	-0.39	0.21
Enterococcus faecalis	673	-20.5	-16.3	-13.1	-34.9	-3.6	-16.94	28.0	-0.36	-0.13
Listeria monocytogenes	754	-17.0	-14.1	-11.7	-26.2	-3.3	-14.23	15.25	-0.12	-0.09
Zymomonas mobilis	189	-18.8	-16.8	-14.7	-28.4	-7.2	-16.89	11.54	-0.29	0.55
Synechocystis sp.	154	-15.58	-13.3	-10.6	-23.8	-5.3	-13.24	13.01	-0.27	-0.08
Streptomyces coelicolor	607	-29.45	-23.7	-14.7	-43.0	-4.2	-22.91	80.12	-0.09	-0.99
Streptomyces venezuelae	548	-30.53	-25.95	-17.9	-43.4	-3.4	-24.45	74.69	0.29	-0.73
Streptomyces tsukubensis	499	-31.65	-25.3	-16.3	-43.0	-3.2	-24.15	84.63	0.2	-1.03
Streptomyces griseus	701	-33.1	-25.9	-15.8	-44.7	-6.1	-24.91	94.04	0.1	-1.18
Streptomyces lividans	471	-28.65	-23.6	-15.45	-42.1	-7.4	-22.68	68.33	0.03	-0.92
Streptomyces clavuligerus	414	-30.1	-25.5	-16.97	-44.1	-3.4	-23.78	72.95	0.26	-0.75
Streptomyces avermitilis	667	-30.5	-25.0	-17.0	-42.4	-3.4	-23.82	73.72	0.19	-0.91

Supplementary Table 4. Results of the two-sided Mann-Whitney U rank test performed for each pairwise comparison of samples on distributions of multiple studied intrinsic terminators features. Statistic - Mann-Whitney U statistic corresponding to the sample A; P-value - associated p-value; FDR - P-value corrected for false discovery rate using the method of Benjamini/Hochberg; FDR < 0.05 - True if FDR < 0.05, False otherwise. [Table provided as separate Excel file, available online]

Supplementary Table 5. Descriptive statistics calculated to characterize distributions of hairpin scores calculated by the TransTermHP pipeline for all identified intrinsic terminators annotated in each dataset. Abbreviations: *nobs* – number of observations; *Q1* – first quartile; *Q3* – third quartile; *min* - minimum; *max* - maximum; *mean* - arithmetic mean; *variance* - unbiased variance, *nobs -1* is used as a denominator; *skewness* – distribution skewness with denominator equal to *nobs*; *kurtosis* - normalized kurtosis of the distribution (kurtosis = 0 for the normal distribution), no degrees of freedom are used for the calculations.

	nobs	Q1	median	Q3	min	max	mean	variance	skewness	kurtosis
Escherichia coli (a)	530	-14.08	-12.4	-10.4	-21.5	-3.0	-12.28	8.35	-0.01	0.36
Escherichia coli (b)	661	-14.0	-12.2	-10.3	-21.5	-3.0	-12.13	8.85	-0.02	0.16
Bacillus subtilis (a)	569	-13.2	-11.2	-9.0	-21.3	-2.6	-11.1	10.26	-0.1	0.07
Bacillus subtilis (b)	1039	-13.2	-11.1	-8.8	-20.9	-2.3	-11.03	11.03	-0.08	-0.08
Bacillus subtilis (c)	885	-13.3	-11.3	-9.1	-22.4	-2.4	-11.28	10.77	-0.09	0.14
Bacillus subtilis (d)	1080	-13.2	-11.2	-8.9	-21.3	-2.4	-11.11	10.8	-0.08	-0.04
Enterococcus faecalis	666	-16.3	-12.7	-9.5	-29.3	-2.4	-12.96	24.53	-0.26	-0.37
Listeria monocytogenes	797	-12.9	-10.4	-8.1	-24.6	-2.2	-10.59	12.96	-0.27	0.01
Zymomonas mobilis	165	-14.7	-13.2	-11.1	-19.8	-2.9	-12.95	8.75	0.04	0.28
Synechocystis sp.	119	-9.8	-7.9	-5.45	-14.6	-2.1	-7.72	8.42	-0.09	-0.72
Streptomyces coelicolor	222	-24.9	-21.45	-18.3	-40.9	-8.1	-21.8	29.07	-0.4	0.75
Streptomyces venezuelae	243	-24.95	-21.4	-18.5	-37.8	-3.1	-21.38	28.85	0.31	0.56
Streptomyces tsukubensis	219	-24.95	-21.4	-17.8	-37.4	-5.6	-21.41	31.81	0.04	0.14
Streptomyces griseus	280	-27.5	-22.95	-19.2	-42.7	-7.4	-23.47	31.9	-0.11	-0.01
Streptomyces lividans	195	-23.75	-20.8	-17.9	-32.4	-8.1	-20.91	21.74	-0.01	0.27
Streptomyces clavuligerus	217	-24.0	-20.7	-18.1	-35.2	-2.1	-20.78	26.87	0.5	1.49
Streptomyces avermitilis	325	-24.0	-20.8	-17.6	-35.1	-4.4	-20.91	26.11	0.02	-0.03

Supplementary Table 6. Descriptive statistics calculated to characterize distributions of hairpin lengths of all identified intrinsic terminators in each dataset. Hairpin length is defined as the number of nucleotides classified as a part of the hairpin loop or stem. Abbreviations: *nobs,* number of observations; *Q1*, first quartile; *Q3*, third quartile; *min*, minimum; *max*, maximum; *mean*, arithmetic mean; *variance*, unbiased variance (*nobs -1* was used as a denominator); *skewness*, distribution skewness with denominator equal to *nobs*; *kurtosis*, normalized kurtosis of the distribution (kurtosis = 0 for the normal distribution), no degrees of freedom were used for the calculations.

	**nobs**	**Q1**	**median**	**Q3**	**min**	**max**	**mean**	**variance**	**skewness**	**kurtosis**
Escherichia coli (a)	691	20.0	24.0	28.5	13	56	24.6	44.97	0.97	1.45
Escherichia coli (b)	957	20.0	25.0	30.0	13	51	25.47	45.97	0.6	0.08
Bacillus subtilis (a)	635	21.0	24.0	27.5	11	48	24.97	31.08	0.97	1.21
Bacillus subtilis (b)	1165	21.0	24.0	28.0	11	54	25.12	33.96	0.91	1.16
Bacillus subtilis (c)	984	21.0	24.0	28.0	11	49	24.97	30.97	1.0	1.43
Bacillus subtilis (d)	1214	21.0	24.0	28.0	11	54	25.1	34.09	1.02	1.66
Enterococcus faecalis	796	27.0	33.0	38.0	12	59	32.86	58.21	0.17	-0.19
Listeria monocytogenes	862	24.0	28.0	33.0	11	57	28.66	48.13	0.76	0.74
Zymomonas mobilis	206	24.0	27.0	32.75	15	59	29.0	54.92	1.31	2.41
Synechocystis sp.	165	23.0	27.0	32.0	12	59	27.52	61.01	0.87	1.52
Streptomyces coelicolor	629	28.0	33.0	38.0	14	58	32.83	44.99	0.05	0.38
Streptomyces venezuelae	573	28.0	33.0	39.0	14	59	33.36	54.89	0.26	0.79
Streptomyces tsukubensis	520	29.0	34.0	39.0	16	57	33.84	49.51	0.0	0.34
Streptomyces griseus	737	29.0	34.0	38.0	14	57	33.4	44.89	-0.1	0.62
Streptomyces lividans	495	28.0	32.0	37.0	16	58	32.38	46.49	0.12	0.28
Streptomyces clavuligerus	435	29.0	35.0	39.0	13	59	34.37	53.36	0.14	0.26
Streptomyces avermitilis	705	28.0	33.0	38.0	16	59	33.01	47.06	0.29	0.63

Supplementary Table 7. Descriptive statistics calculated to characterize distributions of hairpin loop lengths for all identified intrinsic terminators annotated in each experiment and species. Abbreviations: *nobs* – number of observations; *Q1* – first quartile; *Q3* – third quartile; *min* - minimum; *max* - maximum; *mean* - arithmetic mean; *variance* - unbiased variance, *nobs -1* is used as a denominator; *skewness* – distribution skewness with denominator equal to *nobs*; *kurtosis* - normalized kurtosis of the distribution (kurtosis = 0 for the normal distribution), no degrees of freedom are used for the calculations.

	nobs	Q1	median	Q3	min	max	mean	variance	skewness	kurtosis
Escherichia coli (a)	530	4.0	4.0	5.0	3	11	4.4	1.47	1.46	3.12
Escherichia coli (b)	661	4.0	4.0	5.0	3	11	4.38	1.53	1.51	3.24
Bacillus subtilis (a)	569	4.0	4.0	5.0	3	11	4.61	2.27	1.17	1.35
Bacillus subtilis (b)	1039	3.0	4.0	5.0	3	12	4.58	2.37	1.42	2.61
Bacillus subtilis (c)	885	3.0	4.0	5.0	3	12	4.59	2.29	1.29	2.12
Bacillus subtilis (d)	1080	3.0	4.0	5.0	3	12	4.59	2.4	1.4	2.56
Enterococcus faecalis	666	4.0	4.0	6.0	3	13	4.87	2.93	1.4	2.81
Listeria monocytogenes	797	4.0	4.0	6.0	3	12	4.74	2.48	1.29	2.26
Zymomonas mobilis	165	4.0	4.0	5.0	3	10	4.45	1.75	1.06	1.18
Synechocystis sp.	119	3.0	4.0	5.5	3	10	4.61	2.37	1.08	0.92
Streptomyces coelicolor	222	4.0	4.0	5.0	3	11	4.56	2.13	1.45	2.62
Streptomyces venezuelae	243	4.0	4.0	5.0	3	10	4.35	1.53	1.62	3.45
Streptomyces tsukubensis	219	4.0	4.0	5.0	3	12	4.55	2.35	1.67	3.78
Streptomyces griseus	280	4.0	4.0	5.0	3	13	4.55	2.31	2.03	6.1
Streptomyces lividans	195	4.0	4.0	5.0	3	9	4.5	1.78	1.25	1.59
Streptomyces clavuligerus	217	4.0	4.0	5.0	3	10	4.48	2.19	1.66	3.22
Streptomyces avermitilis	325	4.0	4.0	5.0	3	12	4.43	2.27	2.09	5.75

Supplementary Table 8. Descriptive statistics calculated to characterize distributions of tail scores of all identified intrinsic terminators in each dataset. Abbreviations: *nobs*, number of observations; *Q1*, first quartile; *Q3*, third quartile; *min*, minimum; *max*, maximum; *mean*, arithmetic mean; *variance*, unbiased variance (*nobs -1* was used as a denominator); *skewness*, distribution skewness with denominator equal to *nobs*; *kurtosis*, normalized kurtosis of the distribution (kurtosis = 0 for the normal distribution), no degrees of freedom were used for the calculations.

	**nobs**	**Q1**	**median**	**Q3**	**min**	**max**	**mean**	**variance**	**skewness**	**kurtosis**
Escherichia coli (a)	530	-5.5	-4.8	-3.9	-6.4	-2.5	-4.65	0.87	0.39	-0.9
Escherichia coli (b)	661	-5.4	-4.7	-3.8	-6.4	-2.5	-4.58	0.91	0.28	-1.04
Bacillus subtilis (a)	569	-5.6	-5.3	-4.7	-6.4	-2.7	-5.12	0.54	1.16	0.99
Bacillus subtilis (b)	1039	-5.6	-5.3	-4.6	-6.4	-2.5	-5.05	0.63	1.06	0.54
Bacillus subtilis (c)	885	-5.6	-5.3	-4.7	-7.1	-2.6	-5.11	0.56	1.13	0.97
Bacillus subtilis (d)	1080	-5.6	-5.3	-4.6	-6.4	-2.5	-5.04	0.63	1.05	0.51
Enterococcus faecalis	666	-5.7	-5.3	-4.7	-7.0	-2.5	-5.13	0.63	1.0	0.64
Listeria monocytogenes	797	-5.7	-5.5	-5.1	-6.5	-2.9	-5.31	0.37	1.37	2.27
Zymomonas mobilis	165	-5.6	-5.3	-4.4	-6.2	-2.7	-4.95	0.81	0.97	-0.18
Synechocystis sp.	119	-5.6	-5.3	-4.9	-6.2	-3.1	-5.18	0.37	1.06	0.86
Streptomyces coelicolor	222	-3.8	-3.5	-3.1	-5.5	-2.5	-3.52	0.35	-0.52	0.22
Streptomyces venezuelae	243	-4.1	-3.6	-3.1	-5.5	-2.5	-3.59	0.43	-0.57	-0.09
Streptomyces tsukubensis	219	-4.0	-3.5	-3.1	-5.8	-2.5	-3.56	0.47	-0.86	0.58
Streptomyces griseus	280	-3.7	-3.4	-2.9	-5.5	-2.5	-3.43	0.38	-0.77	0.37
Streptomyces lividans	195	-3.8	-3.5	-3.1	-5.6	-2.5	-3.55	0.4	-0.65	0.35
Streptomyces clavuligerus	217	-3.9	-3.5	-3.1	-5.8	-2.5	-3.57	0.5	-1.01	0.7
Streptomyces avermitilis	325	-3.9	-3.4	-3.1	-5.9	-2.5	-3.54	0.44	-0.76	0.38

Supplementary Table 9. statistics calculated to characterize distributions of distances between the hairpin base and the POT for intrinsic terminators identified by the TERMITe in each dataset. Abbreviations: *nobs*, number of observations; *Q1*, first quartile; *Q3*, third quartile; *min*, minimum; *max*, maximum; *mean*, arithmetic mean; *variance*, unbiased variance (*nobs -1* was used as a denominator); *skewness*, distribution skewness with denominator equal to *nobs*; *kurtosis*, normalized kurtosis of the distribution (kurtosis = 0 for the normal distribution), no degrees of freedom were used for the calculations.

	nobs	Q1	median	Q3	min	max	mean	variance	skewness	kurtosis
Escherichia coli (a)	691	3.0	5.0	6.0	0.0	10.0	4.24	3.89	-0.39	-0.32
Escherichia coli (b)	957	3.0	5.0	6.0	0.0	10.0	4.6	4.77	-0.33	-0.12
Bacillus subtilis (a)	635	5.0	6.0	7.0	0.0	10.0	5.42	3.62	-1.14	1.0
Bacillus subtilis (b)	1165	5.0	6.0	7.0	0.0	10.0	5.33	4.17	-0.98	0.47
Bacillus subtilis (c)	984	5.0	6.0	7.0	0.0	10.0	5.58	3.79	-1.16	0.9
Bacillus subtilis (d)	1214	5.0	6.0	7.0	0.0	10.0	5.46	3.78	-1.1	0.73
Enterococcus faecalis	796	4.0	6.0	7.0	0.0	10.0	5.17	5.42	-0.71	-0.35
Listeria monocytogenes	862	5.0	6.0	7.0	0.0	10.0	5.82	3.27	-1.37	1.85
Zymomonas mobilis	206	3.0	5.0	6.0	0.0	10.0	4.48	4.88	-0.47	-0.3
Synechocystis sp.	165	5.0	6.0	7.0	0.0	10.0	5.49	3.63	-0.49	1.25
Streptomyces coelicolor	629	2.0	4.0	6.0	0.0	10.0	4.26	7.71	0.29	-0.7
Streptomyces venezuelae	573	2.0	4.0	6.0	0.0	10.0	3.89	6.99	0.19	-0.8
Streptomyces tsukubensis	520	2.0	4.0	5.0	0.0	10.0	3.71	6.65	0.36	-0.55
Streptomyces griseus	737	2.0	4.0	6.0	0.0	10.0	4.13	7.65	0.17	-0.83
Streptomyces lividans	495	2.0	4.0	6.0	0.0	10.0	4.13	5.96	0.09	-0.64
Streptomyces clavuligerus	435	2.0	5.0	7.0	0.0	10.0	4.91	8.12	-0.05	-1.05
Streptomyces avermitilis	705	2.0	4.0	6.0	0.0	10.0	4.0	6.68	0.26	-0.55

Supplementary Table 10. Descriptive statistics calculated to characterize distributions of the distances between the POT and the nearest upstream gene located on the same strand in each dataset. Abbreviations: *nobs*, number of observations; *Q1*, first quartile; *Q3*, third quartile; *min*, minimum; *max*, maximum; *mean*, arithmetic mean; *variance*, unbiased variance (*nobs -1* was used as a denominator); *skewness*, distribution skewness with denominator equal to *nobs*; *kurtosis*, normalized kurtosis of the distribution (kurtosis = 0 for the normal distribution), no degrees of freedom were used for the calculations.

	**nobs**	**Q1**	**median**	**Q3**	**min**	**max**	**mean**	**variance**	**skewness**	**kurtosis**
Escherichia coli (a)	691	38.0	48.0	69.5	2.0	199.0	62.76	1492.51	1.83	2.65
Escherichia coli (b)	957	38.0	48.0	73.0	1.0	200.0	63.9	1640.91	1.7	2.12
Bacillus subtilis (a)	635	33.0	38.0	46.0	6.0	193.0	46.28	814.43	2.93	9.56
Bacillus subtilis (b)	1165	32.0	38.0	45.0	8.0	196.0	45.03	797.73	2.82	9.19
Bacillus subtilis (c)	984	32.0	38.0	44.0	8.0	196.0	44.13	750.1	3.03	10.56
Bacillus subtilis (d)	1214	31.0	38.0	45.0	8.0	196.0	44.75	824.32	2.81	9.02
Enterococcus faecalis	796	42.0	51.0	71.0	1.0	200.0	62.76	1216.6	1.77	3.12
Listeria monocytogenes	862	37.0	41.0	49.0	1.0	199.0	47.73	676.87	3.29	13.16
Zymomonas mobilis	206	49.0	57.0	70.0	4.0	198.0	66.34	918.05	1.96	4.22
Synechocystis sp.	165	68.0	91.0	126.0	1.0	200.0	98.7	1568.61	0.52	-0.02
Streptomyces coelicolor	629	56.0	76.0	118.0	4.0	200.0	90.29	2121.29	0.78	-0.42
Streptomyces venezuelae	573	53.0	67.0	100.0	1.0	200.0	80.66	1658.47	1.08	0.61
Streptomyces tsukubensis	520	52.0	67.0	100.25	1.0	198.0	79.47	1719.81	0.99	0.33
Streptomyces griseus	737	56.0	69.0	97.0	1.0	200.0	80.42	1494.59	1.04	0.69
Streptomyces lividans	495	52.5	66.0	99.0	7.0	200.0	81.11	1794.79	1.12	0.6
Streptomyces clavuligerus	435	54.0	70.0	111.0	2.0	200.0	84.89	1943.18	0.93	0.1
Streptomyces avermitilis	705	52.0	67.0	99.0	1.0	200.0	79.9	1637.05	1.1	0.63

Supplementary Table 11. Descriptive statistics calculated to characterize distributions of distances between the POT and the nearest downstream gene located on the same strand for all identified intrinsic terminators annotated in each experiment and species. Abbreviations: *nobs* – number of observations; *Q1* – first quartile; *Q3* – third quartile; *min* - minimum; *max* - maximum; *mean* - arithmetic mean; *variance* - unbiased variance, *nobs -1* is used as a denominator; *skewness* – distribution skewness with denominator equal to *nobs*; *kurtosis* - normalized kurtosis of the distribution (kurtosis = 0 for the normal distribution), no degrees of freedom are used for the calculations.

	nobs	Q1	median	Q3	min	max	mean	variance	skewness	kurtosis
Escherichia coli (a)	691	82.0	264.0	1633.0	3.0	40597.0	1469.56	8905260.06	5.59	52.15
Escherichia coli (b)	957	105.0	375.0	1812.0	2.0	40598.0	1704.31	10829139.36	4.69	33.97
Bacillus subtilis (a)	635	82.0	211.0	1305.0	11.0	58833.0	1671.35	20704051.81	7.27	72.94
Bacillus subtilis (b)	1165	95.0	353.0	1697.0	11.0	76739.0	1833.31	21231131.38	8.1	98.32
Bacillus subtilis (c)	984	92.75	325.5	1683.0	11.0	76739.0	1882.39	23045489.27	8.14	97.11
Bacillus subtilis (d)	1214	95.0	351.5	1759.0	11.0	76739.0	1864.88	21777302.75	7.76	90.79
Enterococcus faecalis	794	95.0	282.0	2079.0	2.0	30260.0	2155.74	20104345.95	3.56	14.12
Listeria monocytogenes	862	80.0	234.0	1802.5	7.0	77755.0	2303.26	34549442.77	6.29	56.09
Zymomonas mobilis	206	137.25	425.0	2058.25	5.0	14228.0	1707.09	6762451.13	2.18	4.72
Synechocystis sp.	165	80.0	177.0	1325.0	1.0	8612.0	1125.82	3665028.21	2.35	5.08
Streptomyces coelicolor	629	142.0	732.0	2302.0	1.0	31398.0	1985.13	10383444.1	3.4	17.33
Streptomyces venezuelae	573	84.0	450.0	1834.0	4.0	31480.0	1664.19	9388157.6	3.96	22.85
Streptomyces tsukubensis	519	148.0	558.0	2330.5	6.0	71482.0	2139.07	21523262.55	7.8	98.54
Streptomyces griseus	737	113.0	562.0	1918.0	1.0	68511.0	1907.85	18322104.81	8.62	111.62
Streptomyces lividans	495	100.5	357.0	1784.0	4.0	42700.0	1671.99	11546888.55	5.53	48.7
Streptomyces clavuligerus	435	141.0	484.0	1967.0	4.0	23628.0	1724.57	7674920.0	2.91	12.09
Streptomyces avermitilis	705	108.0	534.0	1927.0	1.0	78901.0	1751.21	16617207.04	11.07	186.94

Supplementary Table 12. Descriptive statistics calculated to characterize distributions of termination efficiencies calculated for all identified intrinsic terminators in each experiment prepared by the protocol published by Mondal et al. (Mondal et al., 2016). Abbreviations: *nobs* – number of observations; *Q1* – first quartile; *Q3* – third quartile; *min* - minimum; *max* - maximum; *mean* - arithmetic mean; *variance* - unbiased variance, *nobs -1* is used as a denominator; *skewness* - skewness with denominator equal to *nobs*; *kurtosis* - normalized kurtosis (kurtosis = 0 for the normal distribution), no degrees of freedom are used for the calculations.

	nobs	Q1	median	Q3	min	max	mean	variance	skewness	kurtosis
Escherichia coli (a)	611	57.6	83.2	95.15	-78.5	100.0	72.75	764.92	-1.19	1.09
Bacillus subtilis (a)	571	66.25	88.6	97.0	-382.4	100.0	76.67	1106.9	-5.51	63.24
Bacillus subtilis (b)	1165	75.2	92.7	98.2	-80.9	100.0	80.89	686.98	-1.94	3.66
Bacillus subtilis (d)	1214	88.52	96.7	99.2	-35.1	100.0	88.69	405.28	-2.92	9.05
